# Squamous Cell Carcinoma Arising in Chronic Osteomyelitis: A Rare Presentation

**DOI:** 10.7759/cureus.49783

**Published:** 2023-12-01

**Authors:** Gopal T Pundkare, Anish Tawde, Kannan A, Nishant Mirchandani, Shubham Lodha

**Affiliations:** 1 Orthopaedics and Traumatology, Bharati Vidyapeeth Deemed University Medical College, Pune, IND; 2 Arthroplasty, KIMS-Sunshine Hospitals, Hyderabad, IND; 3 Orthopaedics, Bharati Vidyapeeth Deemed University Medical College, Pune, IND

**Keywords:** infection, femur, malignant transformation, chronic osteomyelitis, squamous cell carcinoma

## Abstract

Background: Malignant transformation of chronic osteomyelitis is extremely rare. Squamous cell carcinoma (SCC) is the most frequently reported malignancy, with a latency period of 20-50 years after the onset of osteomyelitis.

Case presentation: A 61-year-old man presented with recurrent discharge from the left distal thigh 30 years after open femur fracture. Histopathology showed SCC arising from chronic osteomyelitis with bone invasion. The patient initially declined amputation but eventually consented to transfemoral amputation after symptom recurrence.

Treatment and outcome: Intraoperative frozen section was utilized to determine the level of amputation.

Conclusions: This case highlights the importance of definitive surgical treatment with amputation for SCC arising in chronic osteomyelitis, even after initial patient refusal. Recurrence should prompt the reconsideration of amputation.

## Introduction

Chronic osteomyelitis is characterized by inflammatory destruction and new bone formation in the intramedullary and periosteal areas of the affected bone [1]. In cases of osteomyelitis, the emergence of dead bone signifies a progression to a chronic condition. This dead bone can act as a shelter for bacterial colonies protected by a biofilm, making it difficult for the immune system and antibiotics to effectively combat the infection. Frequently, skin sinus tracts form and can persist for years without surgery, continuously discharging. While these tracts may lessen systemic symptoms, their long-term presence heightens the risk of skin ulcers and potentially the malignant transformation of sinus wall cells [2]. Malignant transformation of osteomyelitis is an extremely rare phenomenon, with squamous cell carcinoma (SCC) being the most frequently reported malignancy [2]. The latency period between the onset of osteomyelitis and diagnosis of SCC ranges from 20 to 50 years [3]. We report a case of SCC arising from chronic osteomyelitis of the femur 30 years after open femur fracture and highlight the importance of definitive surgical treatment with amputation for malignant change in chronic osteomyelitis. Another interesting and unique aspect about the case was that the bone was invaded with tumor and there was no evidence of any mass or ulcer around the sinus which was suggestive of any malignant transformation.

## Case presentation

**Figure 1:**
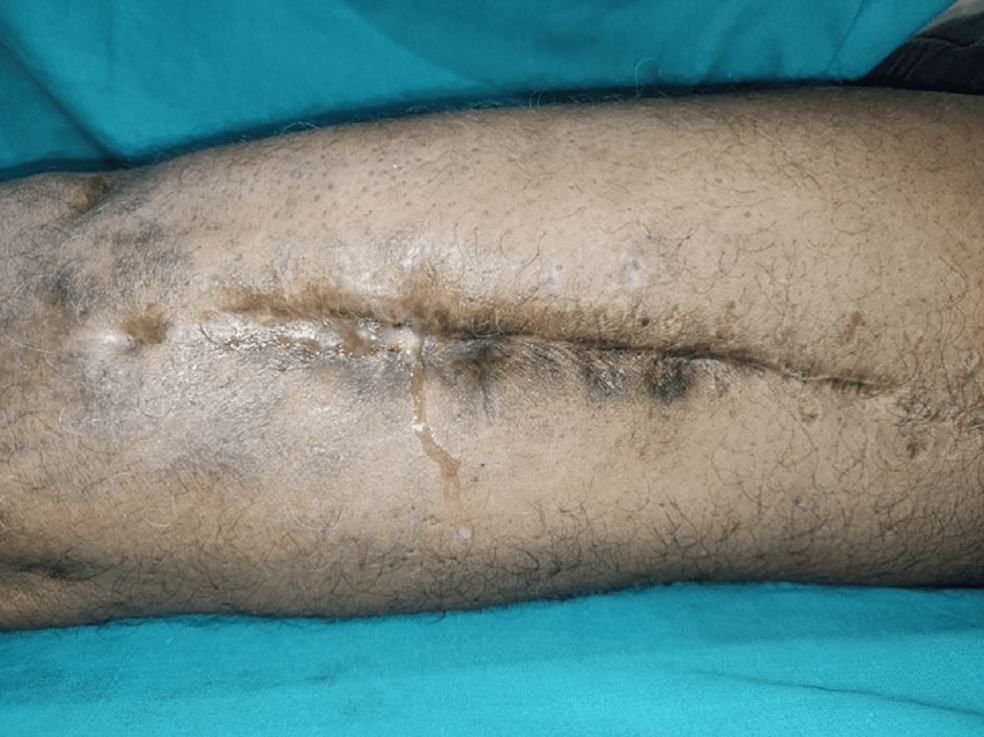
Healed surgical scar with discharging sinus but no palpable mass

A 61-year-old man presented with a 30-year history of pain and intermittent discharge from a discharging sinus on the anterolateral aspect of his left distal thigh (Figure 1). He also had history of on and off fever since many years. His problems started following a road traffic accident and open femur fracture that was treated with open reduction and internal fixation with a plate in 1987. Two weeks postoperatively, he developed wound discharge from the surgical site. The plate was removed in 1988, and he subsequently underwent further two wound debridements. On examination, there was a healed puckered surgical scar with a discharging sinus and local rise in temperature, but no discrete palpable mass (Figure 1). 

**Figure 2:**
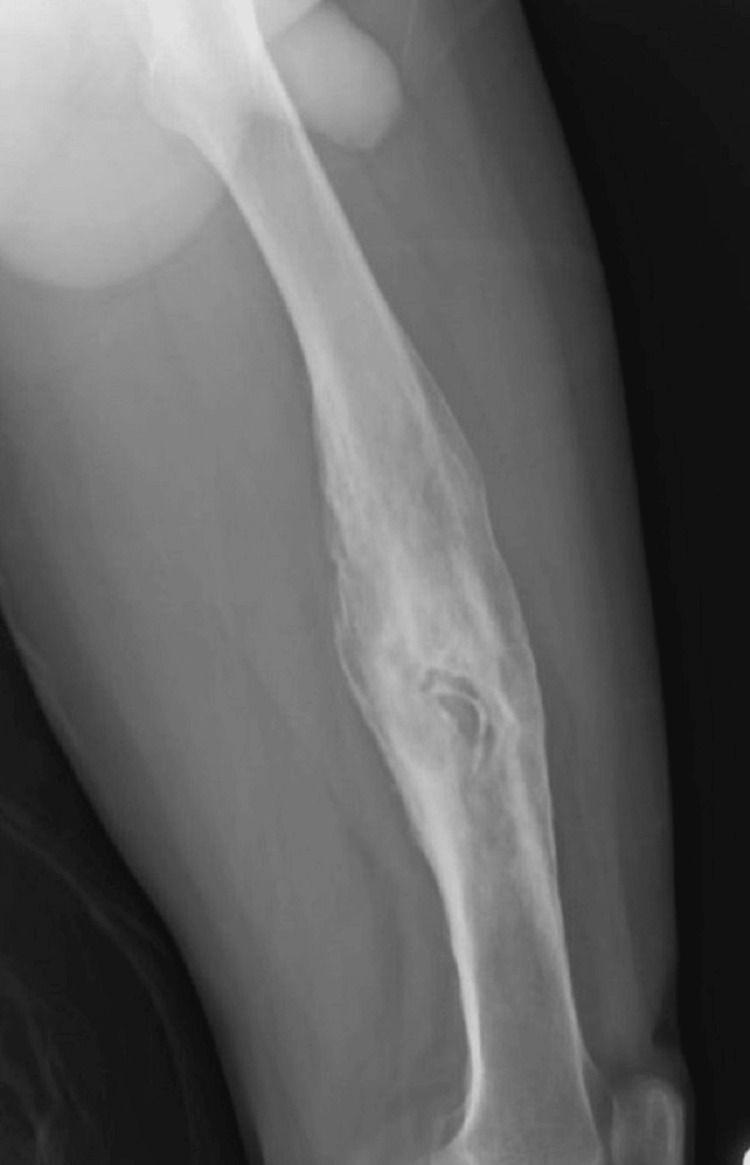
Preoperative radiograph showing the sequestrum surrounded by sclerotic bone

Preoperative radiographs of the patient's femur showed typical sequestrum surrounded by sclerotic bone suggestive of chronic osteomyelitis and involving the midshaft of the left femur (Figure 2).

**Figure 3:**
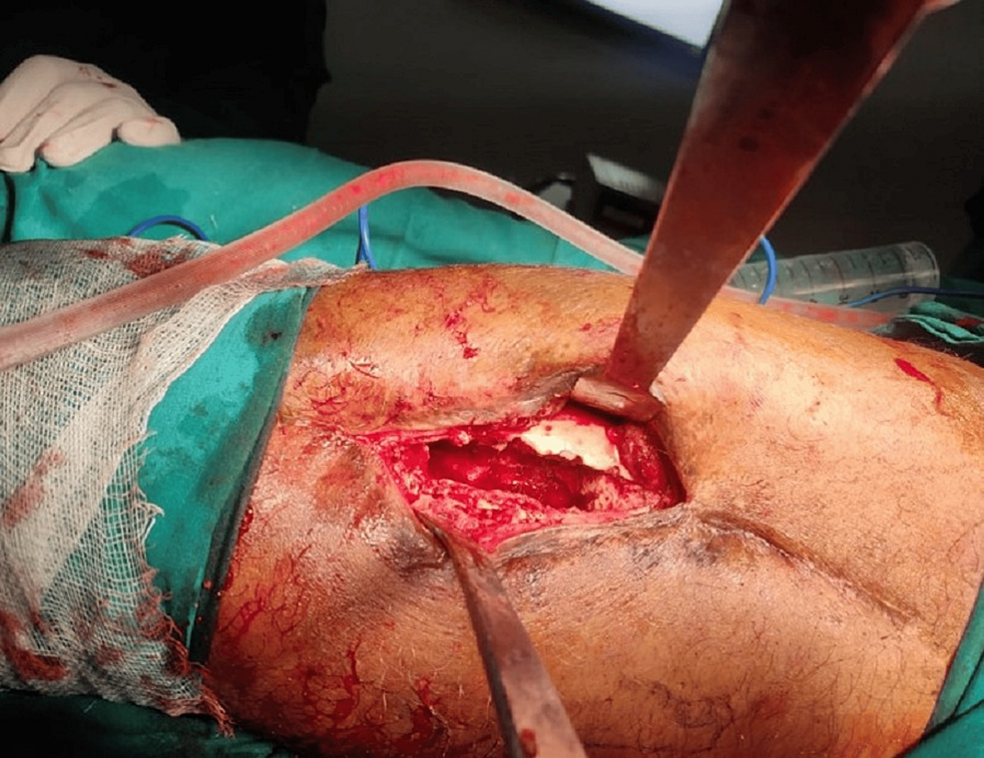
Wound debridement and sequestrectomy

The patient underwent operative debridement with saucerization and sequestrectomy along with the excision of the sinus tract, and antibiotic-coated ceramic granules were placed (Figure 3).

**Figure 4:**
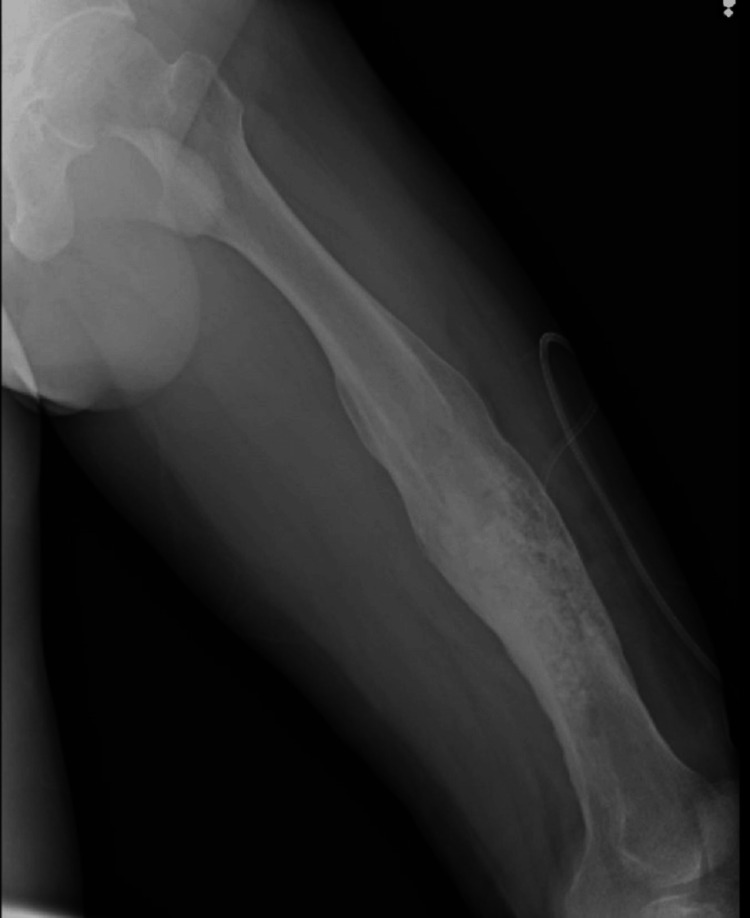
Postoperative radiograph showing the clearance of sequestrum

Postoperative radiograph showed the complete removal of the sequestrum (Figure 4).

**Figure 5:**
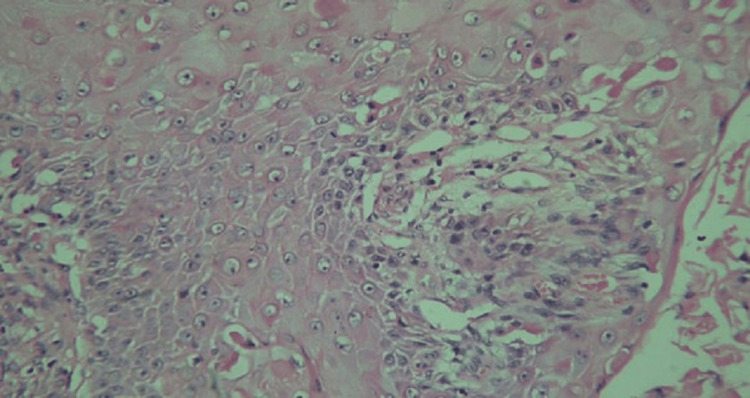
Histopathology showed well-differentiated SCC in a background of chronic osteomyelitis SCC: squamous cell carcinoma

Histopathology showed well-differentiated SCC arising from chronic osteomyelitis, with invasion into the underlying bone (Figure 5). Microbiology revealed *Staphylococcus aureus*.

The patient declined amputation initially but ultimately consented to transfemoral amputation after symptom recurrence 4 months later. Intraoperative frozen section was utilized to ensure adequate margins.

## Discussion

Malignant change should be suspected in cases of chronic osteomyelitis with recent increase in pain, foul-smelling discharge, or bleeding from the sinus [4]. This patient presented with pain and discharging sinus 30 years after open femur fracture, but no discrete mass. Histology confirmed SCC arising from chronic osteomyelitis with bony invasion.

Recent studies and reviews provide insights into the treatment of SCC arising in chronic osteomyelitis. A systematic review conducted on studies published between 1999 and the present day, involving 106 patients, found that amputation was the most commonly used treatment method, being utilized in 81% of cases [2]. This approach was especially favored in patients with metastatic disease, incidental diagnoses at surgery for osteomyelitis, and SCC after pelvic osteomyelitis​​.

Another study, which synthesized cases reported between 1990 and 2019, found that limb amputation was performed in 80.5% of the patients [5]. This study highlighted that despite amputation being the mainstay of treatment, the incidences of local recurrence, metastasis, and SCC-related death remained significant, exceeding 10% in various cases. This study also emphasized close follow-up for patients with local lymphadenopathy at the diagnosis of SCC and those with moderately to poorly differentiated SCC types​​.

A different approach to treatment, Mohs micrographic surgery (MMS), has also been explored as a limb-saving procedure. In a study of four patients with SCC, two of whom had the condition in association with chronic osteomyelitis, MMS was used as a limb-saving procedure for two patients, while the other two underwent amputation [6]. The study suggested that MMS could be a viable option in patients without distant spread of their disease or extensive local disease that could lead to the potential loss of functional stability of the leg postoperative.

These findings indicate that while amputation has been the conventional treatment choice for SCC in chronic osteomyelitis, other options like MMS are being explored and may be suitable in specific cases. However, the choice of treatment depends on several factors, including the extent of the disease, presence of metastasis, and patient-specific considerations.

Definitive treatment is amputation proximal to the tumor or wide local excision, combined with adjuvant chemotherapy and radiation therapy in selected patients [4,5]. Early diagnosis may sometimes allow for treatment consisting of en bloc excision and limb salvage techniques. However, the most effective treatment is prevention with the definitive treatment of the osteomyelitis, including adequate debridement, wide excision of the affected area, and early reconstruction [4]. Amputation was initially recommended for this patient due to bony invasion, but he declined. With inadequate resection, local recurrence is not uncommon, as illustrated by this case [7]. After recurrence of discharge from the operative site four months later, the patient ultimately consented to and underwent transfemoral amputation. Intraoperative frozen section was utilized to ensure adequate margins. The case highlights that definitive surgical treatment with amputation is indicated for SCC arising in chronic osteomyelitis, even when patients are reluctant to undergo the procedure initially.

## Conclusions

This case highlights the importance of definitive surgical treatment with amputation for malignant change in chronic osteomyelitis, even after initial patient refusal. Recurrence of symptoms should prompt the reconsideration of amputation. Intraoperative frozen section helps determine the appropriate level of amputation.
